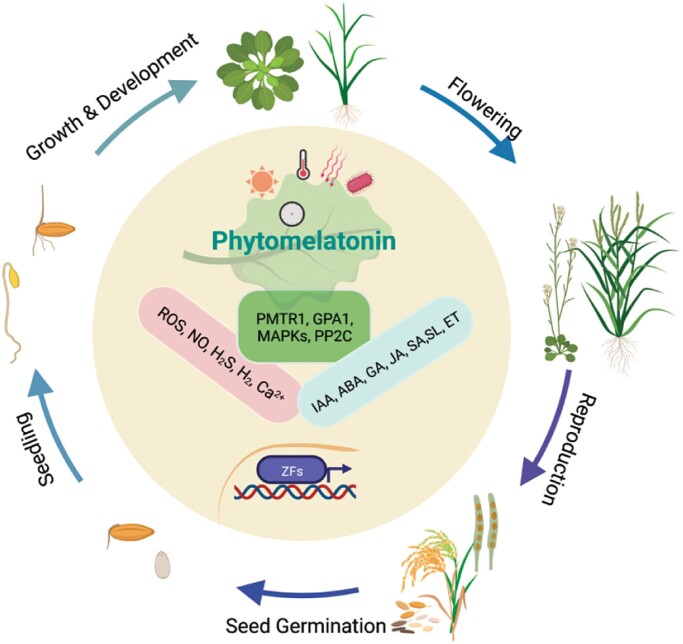# Phytomelatonin: an emerging new hormone in plants

**DOI:** 10.1093/jxb/erac307

**Published:** 2022-09-29

**Authors:** Qi Chen, Marino B Arnao

**Affiliations:** Faculty of Life Science and Technology, Kunming University of Science and Technology, 650500, Kunming, China; Department of Plant Biology (Plant Physiology), School of Biology, University of Murcia, 30100-Murcia, Spain

**Keywords:** Phytomelatonin, phytohormone, signaling pathways, metabolism, nutrient homeostasis, stress resistance

Melatonin (*N*-acetyl-5-methoxytryptamine) synthesized by plants is known as phytomelatonin. It was first detected by several independent research groups in 1995 (Dubbels *et al.*, 1995; Hattori *et al.*, 1995; Kolar *et al.*, 1995; Van Tassel *et al.*, 1995). The biosynthesis of melatonin in animals and plants begins from tryptophan via several similar consecutive enzymatic steps. The first phytomelatonin receptor, PMTR1, was identified in Arabidopsis in 2018 (Wei *et al.*, 2018). Recently, several independent research groups have discovered that PMTR1 and its homologous proteins are required for perceiving phytomelatonin signaling in stomatal closure, seed germination and seedling growth, flowering, leaf senescence, and in responding to various biotic and abiotic stresses in Arabidopsis, tobacco, alfalfa, maize, and cassava (Bai *et al.*, 2022; Chen *et al.*, 2022). Phytomelatonin is therefore comparable to the more well-known plant hormones and regulates nearly all aspects of plant life history (Box 1).

## Metabolism of phytomelatonin

Melatonin is an ancient molecule that is present in nearly all organisms; its structure has not changed in 2.5–3.5 billion years of evolution (Tan *et al.*, 2014). The biosynthesis of melatonin and phytomelatonin begins from tryptophan and shares some similar consecutive enzymatic steps in animals and plants (Arnao *et al.*, 2022; G. Liu *et al.*, 2022; Y. Liu *et al.*, 2022). Plants contain at least six enzymes for phytomelatonin biosynthesis, namely (1) tryptophan decarboxylase (TDC) localized in the cytoplasm, (2) tryptamine 5-hydroxylase (T5H) localized in the endoplasmic reticulum, (3) serotonin *N*-acetyltransferase (SNAT) localized in the chloroplasts, (4) acetylserotonin *O*-methyltransferase (ASMT) and (5) caffeic acid 3-*O*-methyltransferase (COMT), which are both expressed in the cytoplasm, and (6) a putative and unidentified tryptophan hydroxylase (TPH) (Arnao *et al.*, 2022; G. Liu *et al.*, 2022; Y. Liu *et al.*, 2022).

The biosynthesis of phytomelatonin is largely influenced by internal and environmental cues. For example, endogenous phytomelatonin shows circadian rhythms in Arabidopsis, *Chenopodium*, rice, *Paeonia lactiflora*, morning glory, apple, and *Hypericum perforatum* (Chen *et al.*, 2022). Additionally, light, temperature, water status, salt stress, and pathogen invasion can also affect the biosynthesis of endogenous phytomelatonin and expression of related genes (Mou *et al.*, 2022). Several transcription factors can directly bind to the promoters of genes related to phytomelatonin biosynthesis, including heat-shock factor A1a (HsfA1a) in tomato (Cai *et al.*, 2017), RAV1 and RAV2 in the apetala2/ethylene response factor family in cassava (Y. Wei *et al.*, 2018), and ELONGATED HYPOCOTYL 5 (HY5) in Arabidopsis (L. Wang *et al.*, 2022).

In animals, melatonin can be converted into a series of compounds, including cyclic 3-hydroxymelatonin (3-OHM), 2-hydroxymelatonin (2-OHM), and *N*^1^-acetyl-*N*^2^-formyl-5-methoxyamine (AFMK). Interestingly, some enzymes that can degrade melatonin in vertebrates have also been shown to degrade phytomelatonin (G. Liu *et al.*, 2022; Y. Liu *et al.*, 2022). For example, melatonin 3-hydroxylase (M3H) and melatonin 2-hydroxylase (M2H) responsible for the biosynthesis of 3-OHM and 2-OHM have been identified in rice plants. In addition, AFMK has also been identified in *Eichhornia crassipes*, and concentrations of phytomelatonin are significantly decreased in transgenic tobacco plants expressing rice *indoleamine 2, 3-dioxygenase* (ID*O*) (G. Liu *et al.*, 2022; Y. Liu *et al.*, 2022).

## Phytomelatonin signaling in plant growth and development

Signaling pathways and functions are a central research area for phytohormones. The identification of phytomelatonin receptor1 (PMTR1) in 2018 was a turning point in this research field (J. Wei *et al.*, 2018; Arnao and Hernández-Ruiz, 2019; Li *et al.*, 2022). PMTR1 is a putative GPCR-like protein that regulates stomatal closure via either production of reactive oxygen species (ROS) and Ca^2+^ transient influx mediated by GPA1 (G protein α-subunit 1) and NADPH oxidase, or mitogen-activated protein kinase (MAPK) cascades (Chen *et al.*, 2022). Several independent groups have further confirmed that PMTR1 and its homologous proteins are associated with perceiving phytomelatonin signaling in seedling growth, flowering, stomatal closure, immunity, and salt and drought tolerance in Arabidopsis, tobacco, and alfalfa (Chen *et al.*, 2022). More recently, MePMTR1 and ZmPMTR1 have been identified in cassava and maize, respectively (Bai *et al.*, 2022; L.-F. Wang *et al.*, 2022). MePMTR1 and ZmPMTR1 are membrane proteins that show a high affinity for melatonin binding and are required for melatonin-mediated drought resistance and alleviation of darkness-induced leaf senescence and stomatal closure. It is interesting to note that the protein phosphatase MePP2C1 can interact with MePMTR1 and dephosphorylate it at the S11 residue, thereby repressing its binding to melatonin (Bai *et al.*, 2022).

The photoperiod determines plant growth, development, and behaviors. For example, hypocotyl growth and stomatal movements follow a rhythmic pattern, with peak hypocotyl growth and full stomatal closure occurring during the night-time. In Arabidopsis, endogenous phytomelatonin and expression of genes associated with its biosynthesis show 24-h oscillations (Shi *et al.*, 2016; Li *et al.*, 2020), and this is required for daily stomatal closure via a PMTR1-mediated ROS signaling burst in the afternoon (Li *et al.*, 2020). External application of melatonin can induce elongation of hypocotyls and stems, which might be associated with signaling pathways mediated by COP1 (CONSTITUTIVE PHOTOMORPHOGENIC 1), by the G protein α-subunit (Arabidopsis GPA1 and rice RGA1), and by PMTR1 (Chen *et al.*, 2022).

Flowering is a critical stage in plant life history (Mou *et al.*, 2022). Several well known pathways determine the optimum time to flower, including the photoperiod, vernalization, autonomous, age, and gibberellin (GA) pathways (Chen *et al.*, 2022; Mou *et al.*, 2022). Exogenous melatonin delays flowering time in both long- and short-day plants (Chen *et al.*, 2022; Mou *et al.*, 2022). Genetic analyses have shown that the Arabidopsis mutants *comt1* and *snat1* that have lower endogenous phytomelatonin concentrations display earlier flowering times than wild-type Col-0 plants (Zhang *et al.*, 2019). Phytomelatonin delays flowering through interactions with other hormones (eg. GA and strigolactone), ROS signaling, and the central components of floral-gene regulation networks (Chen *et al.*, 2022). CRISPR/cas9-edited plants and overexpressing lines of *PMTR1* show earlier and later flowering, respectively, than Arabidopsis wild-type Col-0 (Yin *et al.*, 2022), indicating the phytomelatonin-mediated delay of flowering time is likely to be dependent on the PMTR1 receptor.

As an essential part of a balanced diet, fruit plays an important role in human nutrition and health. From the perspective of the plant, fruits provide the appropriate environment for the formation and maturation of seeds (Corpas *et al.*, 2022). Endogenous phytomelatonin concentrations vary during the development, growth, and ripening of fruits. For example, in sweet cherry, the phytomelatonin content reaches a maximum at early stages of fruit set and development, and then decreases at the start of ripening (Tijero *et al.*, 2019). External application of melatonin retards senescence and extends the postharvest life of fruits during storage via regulation of ROS, reactive nitrogen species (RNS), and ethylene (Arnao and Hernández-Ruiz, 2020a, 2021; Aghdam *et al.*, 2022; Corpas *et al.*, 2022; K. Wang *et al.*, 2022).

Seed formation, growth, maturation, and germination are crucial developmental stages in the life cycle of seed plants. Several studies have found that melatonin can promote seed germination under stress conditions, especially in old or damaged seeds, improving germination rate probably through fit hormonal balance (K. Wang *et al.*, 2022). In contrast, Lv *et al.* (2021) and Yin *et al.* (2022) have shown that high concentrations of melatonin inhibit Arabidopsis seed germination and seedling growth, possibly due to disorder in ABA, GA, and auxin balance. It is interesting that *PMTR1*-knockout mutants contain higher ABA concentrations in developing seeds, but accumulate lower ABA contents in dry and imbibed seeds than the wild-type Col-0 (Yin *et al.*, 2022), indicating that PMTR1 is involved in phytomelatonin-mediated seed development, dormancy, germination, and seedling growth via crosstalk with ABA signaling. Transgenic Arabidopsis plants overexpressing *AtPMTR1* produce smaller seeds and germinate more slowly than Col-0 plants (Yin *et al.*, 2022). The application of melatonin can induce seedless fruits of pear via regulation of GA pathways (Liu *et al.*, 2018). However, an increase in production of wheat and soybean is observed under abiotic stress conditions when the seeds have been coated with melatonin (Cui *et al.*, 2017; Wei *et al.*, 2015).

## Phytomelatonin functions in nutrient homeostasis and stress resistance

Annual food production needs to be doubled by 2050 to match predicted population growth (Huang *et al.*, 2022); however, at the same time changes in global climate and their impact on current agricultural systems are severely limiting crop growth and yield, and threatening food security. Long-term intensification of agricultural practices and inadequate nutrient inputs have changed the properties of soils, causing mineral nutrient deficiencies and ion toxicities (Sun *et al.*, 2022). Under conditions of nutrient deficiency (e.g. K, Fe, P, S, and N), phytomelatonin is a very important signal molecule in uptake, transport, homeostasis, and use efficiency via regulating the expression of related genes and/or directly regulating activities of transporters, plasma membrane H^+^-ATPase, and the ROS–Ca^2+^ hub, and through crosstalk with other phytohormones (Huang *et al.*, 2022). In addition, phytomelatonin is also required for plant resistance to salinity, osmotic stress, hazardous metals (e.g. Cd, Al, Ni, Pb), drought stress, and high-light stress via receptor-dependent or -independent systems in the regulation of ROS, RNS, and H_2_ signaling and in crosstalk with other plant hormones (e.g. auxin, cytokinin, ABA, ethylene, brassinosteroids, jasmonates, salicylic acid, and strigolactones; Arnao and Hernández-Ruiz, 2020b; Sun *et al.*, 2022; Y. Wang *et al.*, 2022).

Phytomelatonin has been suggested to be a central molecule in plant resistance to bacterial, fungal, and viral diseases via crosstalk with ROS, RNS, and phytohormones (e.g. salicylic acid, jasmonates, ABA) and via regulation of expression of genes associated with pathogen responses, disease resistance, and immunity (Zeng *et al.*, 2022). In *Panax notoginseng*, foliar application of melatonin decreases the incidence of leaf diseases such as grey mould, round spot, and black spot, with phytomelatonin-induced stomatal closure playing a prominent role in preventing bacterial invasion in Arabidopsis and *P. notoginseng* via PMTR1-medaited activation of the GPA1- and MAPK-signaling pathways, respectively (Yang *et al.*, 2021). It is worth noting that *pmtr1* and *gpa1* mutants are also insensitive to flg22-induced stomatal closure in Arabidopsis, indicating that PMTR1 is required for FLS1- and BAK1-mediated flg22 signaling in stomata-mediated defence (Yang *et al.*, 2021).

## Phytomelatonin regulates primary and secondary metabolism

Using light energy, photosynthesis converts carbon dioxide and water into organic compounds and oxygen, in a process that is essential for all living organisms on our planet. In plants, ROS are by-products of O_2_ during the photosynthetic and oxidative phosphorylation processes in chloroplasts, where phytomelatonin is mainly synthesized. The biosynthesis of phytomelatonin can be induced by light, and shows daily rhythms and peaks during daytime (Li *et al.*, 2020; Chen *et al.*, 2022; G. Liu *et al.*, 2022; Y. Liu *et al.*, 2022), which may play an important role in scavenging ROS when it is overproduced. As well as directly protecting chloroplasts from excess ROS stress, phytomelatonin also acts as a signaling molecule in regulating chlorophyll and protein synthesis and degradation, photosynthetic rates, and the metabolism of sugars, lipids, and secondary metabolites through modulating the transcription of related genes and crosstalk with hormone signals (Arnao *et al.*, 2021, 2022; Lee *et al.*, 2022; Yang *et al.*, 2022).

Secondary metabolites are essential for plant defence and hence crop yield. Application of exogenous melatonin can regulate the levels of phenolic compounds, glucosinolates, and terpenoids (Arnao *et al.*, 2022). In this special issue, Zhao *et al.* (2022) show that applied melatonin and endogenous phytomelatonin enhance stem strength in herbaceous peony and tobacco plants by increasing lignin content and the S/G lignin compositional ratio. Phytomelatonin might have dual roles in the biosynthesis of flavonoids; for example, exogenous melatonin can induce flavonoid biosynthesis under stress, after harvest, and in conditions of leaf senescence and endogenous phytomelatonin plays a negative role in the biosynthesis of flavonoids under normal growth conditions (Chen *et al.*, 2022). For instance, melatonin increases luteolin biosynthesis and up-regulates the expression of related genes in salt-stressed pigeon pea plants (Song *et al.*, 2022), which might be associated with maintenance of antioxidant capacity under stress conditions (Chen *et al.*, 2022). However, exogenous melatonin and endogenous phytomelatonin have a comparable negative effect on the biosynthesis of anthocyanins in Arabidopsis, rice, and alfalfa (Chen *et al.*, 2022).

## Conclusions and future directions

During the past two decades, at least 1000 articles have been published about the physiological functions of phytomelatonin. The number of such studies is expected to increase in the coming years (Arnao *et al.*, 2022), indicating that phytomelatonin is now a very active research area in plant science.

Plant hormones are chemical compounds present in very low concentrations that act at or near the site of synthesis, or in distant tissues to regulate all aspects of plant growth, development, and environmental responses (Santner *et al.*, 2009). Biosynthesis pathways of phytomelatonin have been well characterized in many plant species (Arnao *et al.*,2022; G. Liu *et al.*, 2022; Y. Liu *et al.*, 2022). The identification of the phytomelatonin receptor PMTR1 in Arabidopsis and its homologous proteins in alfalfa, tobacco, maize, and cassava should lead to exciting models of the perception and functioning of this new phytohormone (Bai *et al.*, 2022; Chen *et al.*, 2022). Phytomelatonin is distributed in almost all tissues (e.g. roots, stems, leaves, flowers, fruits, and seeds) and is involved in nearly all physiological processes (Box 1), and several phytomelatonin-mediated functions are dependent on PMTR1, including seed development and germination, shoot growth, flowering, fruit development and ripening, stomatal closure, and biotic and abiotic stresses (Chen *et al.*, 2022).

Although phytomelatonin is becoming well established as a new plant hormone (Arnao and Hernández-Ruiz, 2019; Chen *et al.*, 2022; Li *et al.*, 2022; J. Wei *et al.*, 2018), there are many issues that remain to be resolved.

(1) Phytomelatonin transport. Phytomelatonin is structurally similar to auxin, which need various types of transporters for distribution to the respective basal parts after being synthesized in the apices of shoots and roots. In animals, the glucose transporter GLUT1 and the oligopeptide transporters PEPT1/2 have been proposed to transport melatonin across plasma and mitochondrial membranes. Local and long-distance transport of phytomelatonin may have an essential role in many aspects of plant growth and development, and in stress responses. Identification of phytomelatonin transporters in future studies should help uncover the mysteries of this new phytohormone.
(2) Phytomelatonin receptors and signaling transduction pathways. Although several studies have found that PMTR1 plays important roles in perceiving phytomelatonin in plant growth and development, and in stress resistance, examination of the underlying signal-transduction pathways is still a nascent field. For example, do plants contain other phytomelatonin receptors? How does PMTR1 regulate the G protein and MAPK cascades? Are there any transcription factors involved in the phytomelatonin-mediated expression of large numbers of genes?
(3) The use of phytomelatonin in agriculture. Exogenous melatonin affects a broad range of physiological processes and has been shown to have significant potential in agricultural practices; for example, in improving photosynthesis and crop production, in delaying flowering time and extending the postharvest life of fruit, in regulating seed development and germination, and in increasing contents of valuable secondary metabolites. In addition, the application of natural phytomelatonin-rich plant extracts as alternative growth regulators for crops is an interesting area of study (Hernández-Ruiz *et al.*, 2021). The cultivation of crops with modified endogenous phytomelatonin concentrations or signaling components via the use of current plant molecular and genetic methods might also be in the subject of future studies.

**BOX 1. Phytomelatonin signaling in the plant life cycle.**

Melatonin (*N*-acetyl-5-methoxytryptamine) synthesized by plants is called phytomelatonin. Its biosynthesis begins from tryptophan and can be regulated by various internal (e.g. circadian clock, phytohormones) and external cues (e.g. light, high or low temperature, water and nutrient status, pathogen invasion). Phytomelatonin affects nearly all aspects of the plant life cycle, from seed germination, seedling establishment, growth and development, to flowering and fruit ripening, and it is also involved in stress responses involving the regulation of GPA1, MAPKs, ROS, RNS, gasotransmitters signals, and crosstalk with other plant hormones, which may occur via the PMTR1 phytomelatonin receptor.